# Severe Vitamin K-dependent Coagulopathy from Rodenticide-contaminated Synthetic Cannabinoids: Emergency Department Presentations

**DOI:** 10.5811/westjem.2021.2.46317

**Published:** 2021-07-15

**Authors:** Erik Wright, John W. Hafner, Gregory Podolej, Douglas L. Feinstein, Richard van Breemen, Israel Rubinstein, Steven Aks, Michael Wahl

**Affiliations:** * University of Illinois College of Medicine Peoria, OSF Saint Francis Medical Center, Department of Emergency Medicine, Peoria, Illinois; † University of Illinois College of Medicine Peoria, Department of Emergency Medicine, Peoria, Illinois; ‡ University of Illinois Chicago, Jesse Brown VA Medical Center, Department of Anesthesiology, Chicago, Illinois; § Oregon State University, Linus Pauling Institute, Department of Pharmaceutical Sciences, Corvallis, Oregon; ¶ University of Illinois Chicago, Jesse Brown VA Medical Center, Division of Pulmonary, Critical Care, Sleep, and Allergy Medicine, Chicago, Illinois; || Rush University, Department of Emergency Medicine, Chicago, Illinois; # John H. Stroger Hospital of Cook County, Department of Emergency Medicine, Chicago, Illinois

## Abstract

**Introduction:**

Synthetic cannabinoids are a rapidly expanding subset of designer drugs widely available in the United States since 2008. In Illinois during the spring of 2018, over 160 documented cases of bleeding and prolonged coagulopathy occurred secondary to contaminated synthetic cannabinoids.

**Methods:**

We conducted a retrospective cohort study consisting of 38 patients to describe the initial emergency department (ED) presentation, diagnosis, and treatment.

**Results:**

Through serum testing we found that three long-acting anticoagulant rodenticides (LAAR) were detected in patients who had inhaled these tainted products: brodifacoum, difenacoum, and bromodialone.

**Discussion:**

This study encompasses the largest ED presentation of LAAR poisoning via the inhalational route known to date.

**Conclusion:**

The emergency physician should be aware of the potential for tainted coingestants as the cause of undifferentiated coagulopathy.

## INTRODUCTION

Synthetic cannabinoids have become a widely used type of designer drug in the global drug market.^1^ Synthetic cannabinoids first made their appearance in the United States in 2008 and are sold under numerous names including “K2,” “Spice,” and “Black Magic.” These drugs have long evaded law enforcement due to the drug manufacturers’ ability to quickly alter chemical formulas and generate novel products that have yet to be made illegal under the Controlled Substances Act. In addition, most of these are packaged and sold as herbal products and labeled as “not for human consumption” to further circumvent drug laws.^2^

After being dissolved in solvent, synthetic cannabinoids are typically formulated and sprayed onto an herbal product that is then smoked and inhaled.^3^ A wide array of symptoms has been associated with ingestion from these compounds. While some users report similar euphoric effects to that of marijuana, there have been other significant adverse reactions reported. Most common adverse symptoms reported include paranoid delusions, psychosis, supraventricular tachycardia, seizures, and altered sensorium.^4^ Additionally, there are many reports describing associations of synthetic cannabinoids with acute medical conditions including ischemic and hemorrhagic strokes, thrombotic microangiopathy, disseminated intravascular coagulation, immune thrombocytopenic purpura, rhabdomyolysis, and death.^5^–^9^

Illicit drugs are often adulterated with other products to increase profits and/or to enhance or alter the drugs’ effects on the body. Several substances including both legal and illegal compounds have been used to achieve these effects.^10^ Interestingly, there are numerous case reports surrounding the use of warfarin as an adulterant “lacing compound.”^11^–^15^ The addition of warfarin or long-acting anticoagulant rodenticides (LAAR) may alter CYP P450 metabolism of the psychoactive compound and act to enhance the high. We surmise drug manufacturers and distributors have exploited this pharmacological interaction in the past based on several other reported cases.

During the spring of 2018, a large influx of patients presented to area hospitals in Illinois with unfounded coagulopathy and bleeding. The outbreak began in mid-March 2018 with over 160 reported cases in Illinois across 15 counties through June 2018.^16^ Through July 2018 the number of cases increased to 255 with eight reported deaths.^17^ By the end of July, over 55% of the documented cases had occurred in Peoria, Tazewell, and surrounding counties in Illinois. Due to symptoms of significant, prolonged bleeding and lack of known exposure to vitamin K_1_ antagonists there was concern that these patients had been inadvertently exposed to a long-acting anticoagulant. A large, interdisciplinary task force composed of members of the US Centers for Disease Control and Prevention, Illinois Poison Center, Illinois Department of Public Health, law enforcement agencies, and health departments was convened to elucidate the cause of this unexplained coagulopathy. It was promptly discovered that poisoned patients had been exposed to tainted synthetic cannabinoids that largely tested positive for brodifacoum, a LAAR.^18^

Other researchers have described a similar population at a single Illinois academic center.^19^,^20^ While those studies concentrated on the inpatient population, treatment and long-term therapy, our focus is to address the emergency department (ED) presentation, diagnosis, and treatment. While the populations are similar, we feel the difference in focus is substantive as the emergency physician is tasked with diagnosis, stabilization, and treatment initiation prior to the patient’s hospital stay. Our goal is to help readers recognize and diagnose patients suffering from bleeding diathesis in the ED as well as to identify potential resuscitative treatment strategies via descriptive data from a recent LAAR outbreak.

**Population Health Research Capsule**

What do we already know about this issue?

*An outbreak of bleeding diathesis in Illinois in spring 2018 was linked to exposure to synthetic cannabinoids contaminated with long-acting anticoagulant rodenticides*.

What was the research question?

*To elucidate management therapies we investigated patients who presented to our ED with concerns for exposure to anticoagulants*.

What was the major finding of the study?

*Many of our patients required active reversal of anticoagulant effects with Vitamin K and/or fresh frozen plasma, and a high number were admitted to the intensive care unit*.

How does this improve population health?

*The emergency physician must be prepared for and aware of the possibility of future outbreak linked to tainted synthetic cannabinoids*.

## METHODS

### Study Design

We conducted a retrospective cohort study to describe the initial ED presentation, diagnosis, and treatment of inhaled LAAR-induced coagulopathy.

### Population

This study was conducted at two Illinois academic urban EDs with annual patient visits of approximately 85,000 and 120,000, respectively. We performed chart review of all patients with suspected brodifacoum-related coagulopathy from contaminated synthetic cannabinoids presenting to the ED. Patients with reported exposure who presented to either of these ED between March 29–April 23, 2018 were included in this study. Patients were identified from internal and public health registries, from patients themselves self-identifying as having an exposure, or who were identified by hospital providers as having an exposure. Using defined variables, we abstracted ED and hospital charts, and all data was deidentified prior to analysis. The institutional review boards (IRB) of the University of Illinois College of Medicine at Peoria and Oregon State University reviewed and approved this study prior to initiation. All data remained deidentified throughout.

### Samples

Serum samples were obtained from 38 patients from Illinois academic urban EDs. Initial blood samples were obtained for clinical care of these patients. Leftover serum from clinical draws was then placed in vacutainers and stored at −80°C until analysis. Samples were sent to and analyzed at the Linus Pauling Institute, Corvallis, OR. Ultra performance liquid chromatography - tandem mass spectrometry (UHPLC-MS/MS) analysis was used to quantify plasma concentrations of brodifacoum, difenacoum and bromadiolone, three structurally distinct LAARs.

### Statistics

We used descriptive statistics to describe the population, define common symptomatology, and identify successful treatment regimens. Unadjusted linear regression analysis was used to describe relationships between plasma LAAR levels and international normalized ratio (INR) values. We used Pearson coefficients to investigate the correlation between variables.

## RESULTS

**Figure 1:**
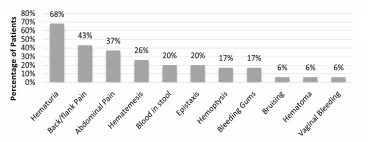
Thirty-eight patients with identified exposure to super warfarin-tainted synthetic cannabinoids.

A total of 38 patients met criteria for inclusion in this study. Of the patients included, 24 males (68%) and 14 females (36%) were identified as being exposed to tainted synthetic cannabinoids. Ages ranged between 23–65 years with a mean age of 37 years at time of presentation. Of these patients, 76% (n = 24) were identified as White. This cohort experienced high admission rates to the hospital with 92% of patients (n = 35) being admitted. The three patients not admitted to the hospital left the ED against medical advice (AMA). Mean length of stay for those admitted was 4.1 days, with a range of 1–11 days. Readmission rates were also very high for this group as 30% of patients (n = 12) were readmitted within 30 days of their initial presentation. Among the wide variety of presenting symptoms the most common presenting complaint was back and or flank pain and the most common site of bleeding was from the urinary tract (Figure 1).

On average, patients had significantly elevated INR values at time of presentation. The INRs ranged from 1 to >20. (The maximum upper limit of on-site laboratory testing is an INR level of >20.) The mean INR at presentation was 14.5. At time of discharge from the hospital, the mean INR was 2.5.

**Figure 2:**
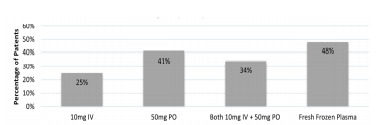
Thirty-eight patients presented and were identified as being exposed to tainted synthetic cannabinoids. Initial emergency department treatment included oral vitamin K1, IV vitamin K1, and fresh frozen plasma. *IV*, intravenous; *PO*, by mouth; *mg*, milligram.

**Figure 3:**
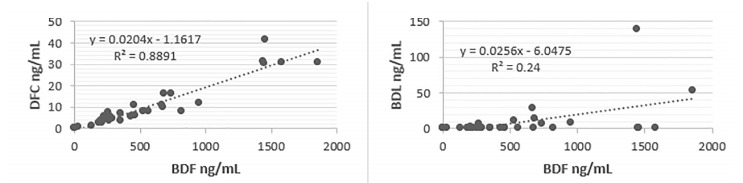
Left: Serum brodifacoum levels plotted on X axis; serum difenacoum levels plotted on Y axis. Linear relationship indicates likely co-contaminant. Right: Serum brodifacoum levels plotted on X axis; serum bromadiolone levels plotted on Y axis. Bromadiolone levels were around 4% of brodifacoum levels, suggesting this is a minor metabolite or a minor co-contaminant. *ng*, nanogram; *mL*, milliliter.

Reversal of LAAR-related coagulopathy was at the treating physician’s discretion. Several therapeutic decisions were made in consultation with Illinois Poison Control. Patients were treated with a combination of oral vitamin K_1_, intravenous (IV) vitamin K_1_, and fresh frozen plasma (FFP). Two patients left AMA before being treated. Of those treated, 25% (n = 9) received 10 milligrams (mg) IV vitamin K_1_; 41% (n = 16) received 50 mg oral vitamin K_1_ as monotherapy, and 34% (n = 13) received a combination of 50 mg oral vitamin K_1_ and 10 mg IV vitamin K_1_. In addition to vitamin K_1_, 48% of patients (n = 18) also received FFP with a dose range of 1–4 units while in the ED (Figure 2). There were no patients treated solely with FFP, which was always used in conjunction with vitamin K_1_ therapy. Brodifacoum, difenacoum, and bromodialone were detected in serum samples. Brodifacoum and difenacoum were detected in 37/38 samples (97%), and bromadiolone was detected in 24/38 samples (63%). Brodifacoum was the predominant LAAR detected; however, it appears that given the high prevalence of difenacoum and its strong correlation to brodifacoum levels (Figure 3) that difenacoum was a co-contaminant of the synthetic cannabinoids, or possibly a minor breakdown product of brodifacoum. In contrast, bromadiolone was detected in few samples overall and had a weaker correlation with brodifacoum levels (Figure 3). This may suggest that only some batches of synthetic cannabinoids were co-contaminated with bromadiolone, or it could reflect the contaminant’s more rapid metabolism compared to the other LAARs.

**Figure 4:**
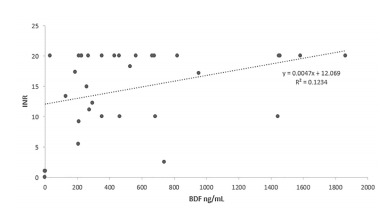
Serum brodifacoum levels plotted on X axis, INR plotted on Y axis. There is a linear correlation with respect to increasing serum brodifacoum levels and elevated INR. This likely is in part due to the volume of distribution into tissues. *BDF*, brodifacoum; *INR*, international normalized ratio; *ng*, nanogram; *mL*, milliliter.

Levels of LAAR and serum INR correlated in a weakly linear fashion (Figure 4). These LAARs have significant distribution into tissues and sequester in the liver; therefore, serum levels of LAAR do not fully represent total body accumulation and may account for at least part of the significant variability of brodifacoum levels and INR.^21^

## DISCUSSION

In the largest cohort of inhalational LAAR coagulopathy to date, many of the patients were quickly recognized and triaged in the ED. The initial treatments in the ED focused on recognition and stabilization as well as reversal of their coagulopathy. The most prominent presenting symptoms included complaints of back and/or flank pain and abdominal pain. Physical manifestations of coagulopathy, including hematuria, bloody stools, and epistaxis and mucosal bleeding, were also observed. Although the exact reasons for combining, or tainting, synthetic cannabis with LAAR is unknown, it is hypothesized that potentiation of cannabinoid effects may have been the desired outcome.^22^–^25^ Regardless of intent, recognizing the potential of contamination of street drugs is extremely salient to the emergency physician. Since the Illinois outbreak that occurred between March-May 2018, there have been further outbreaks of tainted synthetic cannabinoid coagulopathy throughout the East Coast.^26^ As this would suggest, the outbreak in Illinois does not appear to be an isolated incident, and continued vigilance and awareness of this ongoing problem by emergency care providers is necessary.

This cohort of patients was largely treated with vitamin K_1_ in both oral and IV formulations while in the ED. Early involvement of Illinois Poison Control allowed for additional treatment recommendations and appropriate surveillance of the outbreak. Most patients were given either 50 mg oral vitamin K_1_ and/or 10 mg IV vitamin K_1_. However, in those with more significant bleeding, FFP in doses between 1 to 4 units was also used. More advanced products such as Kcentra and factor eight inhibitor bypassing activity (FEIBA) were not used. These products have been shown in several studies to rapidly reverse LAAR-induced coagulopathy and are recommended for those with life-threatening bleeding.^27^–^28^

Treatment for LAAR-induced coagulopathy outside the initial ED stay has proven to be difficult. Many of these patients have experienced repeat ED visits with 30% readmitted in the first 30 days. Many patients were sent home with high doses of oral vitamin K_1_, ranging from 50–150 mg daily. With 15 mg of generic vitamin K_1_ estimated to cost around 80 US dollars, this treatment was often cost-prohibitive for many patients. We suspect cost was the reason many patients with coagulopathy went untreated and suffered from recurrent bleeding, comorbidities, and repeat hospitalizations. Additionally, the pharmacokinetics of brodifacoum (which has a half-life up to 40 hours) can cause patients to suffer from coagulopathy for up to 12 months post ingestion. Until this time, many experts recommended using serum INR to guide vitamin K_1_ therapy for patients with ingestions. With data from these outbreaks, new proposals suggest that following LAAR levels may be the best way to determine when vitamin K_1_ therapy may be stopped.^29^,^30^

## LIMITATIONS

This descriptive study of the largest inhalational LAAR poisoning to date is not without limitations. First is that we conducted a retrospective chart review of patient data. While this study is limited by the standard biases that retrospective chart reviews suffer, we have addressed some of these aspects. During this outbreak, IRB approval was obtained to allow for a prospective approach to standard documentation; this limited some of the collection discrepancies. In addition, prior to chart review we created a standardized abstraction form allowing for a systematic approach to data retrieval. Secondly, while this is the largest tainted inhalational LAAR cohort to date, inherently the patient population is limited. Although we were able to formulate some correlations given the sample size, results may be more pronounced with a larger cohort.

This is the largest cohort of inhalational LAAR toxicity known to date. Recurrences of smaller outbreaks would suggest that LAAR-contaminated synthetic cannabinoids may not be isolated to synthetic cannabinoids.

## CONCLUSION

Working on the frontlines of healthcare, the emergency physician should be aware of the potential for tainted coingestants as the cause of undifferentiated coagulopathy. Long-acting anticoagulant rodenticide poisoning can usually be treated with vitamin K_1_, with the majority of these patients needing long-term outpatient treatment. For those with life-threatening bleeding more advanced products including fresh frozen plasma, Kcentra and FEIBA may be indicated. Additionally, the emergency physician should be aware of the high potential for return visits in these patients for recurrent bouts of coagulopathy due to the prolonged course of action of the drug.
